# Supplementary material for: A conserved neuropeptide system links head and body motor circuits to enable adaptive behavior
Source: eLife. 2021 Nov 12;10:e71747. doi: 10.7554/eLife.71747 (PMC8626090; doi:10.7554/eLife.71747)
Supplement: Supplementary file 1. [file elife-71747-supp1.docx]

**Supplementary File 1**

Strains generated/used in this work

| **Strain** | **Genotype** | **Strain information** |
| --- | --- | --- |
| IZ908 | *nlp-12(ok335) I* | outcrossed from RB607 (CGC) |
| IZ2287 | *ckr-1(ok2502) I* | outcrossed from RB1923 (CGC) |
| LSC0032 | *ckr-2(tm3082) III* | outcrossed from Japanese knockout allele |
| IZ2304 | *ckr-1(ok2502) I; ckr-2(tm3082) III* |  |
| IZ1152 | *ufIs104* | *Pnlp-12::nlp-12::nlp-12 3’UTR (100 ng/µL), Plgc-11::GFP [pHP6 (50 ng/µL)]*  *1.76 kb PCR product containing the nlp-12 promoter and genomic locus (−354 bp to +1407 bp relative to the transcriptional start).* |
| IZ1284 | *ckr-1(ok2502) I; ufIs104* |  |
| IZ1231 | *ckr-2(tm3082) III; ufIs104* |  |
| IZ1295 | *ckr-1(ok2502) I; ckr-2(tm3082) III; ufIs104* |  |
| IZ2544 | *lin-15(n765ts); ufEx942* | *Pckr-1::ckr-1::SL2::GFP [pRB25 (50 ng/µL)]*  *+ Plin-15::lin-15+[pL15EK (50ng/µL)]* |
| IZ1908 | *ufIs141* | *pCKR-1::CKR-1::SL2::mCherry*  *[pDT205 (40 ng/µL)]* |
| IZ2065 | *ufIs148* | *Pckr-2::GFP [pDT195 (20 ng/µL)]* |
| IZ2251 | *ufIs141; ufIs148* | *Pckr-1::ckr-1::SL2::mCherry*; *Pckr-2::GFP* |
| IZ2633 | *ufEx942; ufIs43* | *Pckr-1::ckr-1::SL2::GFP, Pacr-2::mCherry [pPRB6 (30ng/µL)]* |
| IZ2280 | *ufIs141; vsIs48* | *Pckr-1::ckr-1::SL2::mCherry; Punc-17::GFP* |
| IZ2468 | *ufIs141; akEx263* | *Pckr-1::ckr-1::SL2::mCherry; Punc-4::GFP* |
| IZ2245 | *ufIs141; oxIs12* | *Pckr-1::ckr-1::SL2::mCherry; Punc-47::GFP* |
| IZ2246 | *ufIs141; kyIs51* | *Pckr-1::ckr-1::SL2::mCherry; Podr-2(2b)::GFP* |
| IZ2454 | *ufIs141; ufEx863* | *Pckr-1::ckr-1::SL2::mCherry; Podr-2(18)::GFP* |
| IZ2248 | *ufIs141; mgIs18* | *Pckr-1::ckr-1::SL2::mCherry; Pttx-3::GFP* |
| IZ2249 | *ufIs141; oyIs18* | *Pckr-1::ckr-1::SL2::mCherry; Pgcy-8::GFP* |
| IZ2250 | *ufIs141; oyIs14* | *Pckr-1::ckr-1::SL2::mCherry; Psra-6::GFP* |
| IZ2447 | *ufIs141; otIs337* | *Pckr-1::ckr-1::SL2::mCherry; Plad-2::GFP* |
| IZ2499 | *ufIs141; zfIs6* | *Pckr-1::ckr-1::SL2::mCherry; Plgc-55::GFP* |
| IZ3533 | *ufIs141; ufEx1485* | *Pckr-1::ckr-1::SL2::mCherry ;*  *Podr-2(16)::GFP[pNB60 (50 ng/µL)]; Punc122::GFP (50ng/µL)* |
| IZ3591 | *ufis141; ufEx1504* | *Pckr-1::ckr-1::SL2::mCherry ; Pflp-22∆4::GFP [pSR17 (50ng/µL)]; Punc-122::RFP (50 ng/µL)* |
| IZ2635 | *ufEx942; dbEx721* | *Pckr-1::ckr-1::SL2::GFP, Pnpr-4::RFP* |
| IZ2455 | *ufIs141; adEx1616* | *Pckr-1::ckr-1::SL2::mCherry; Pser-4::GFP* |
| IZ2459 | *ufIs141*; *njIs10* | *Pckr-1::ckr-1::SL2::mCherry; Pglr-3::GFP* |
| IZ2504 | *ufIs141; otIs123* | *Pckr-1::ckr-1::SL2::mCherry; Psra-11::GFP* |
| IZ2546 | *ufIs141; mgIs42* | *Pckr-1::ckr-1::SL2::mCherry; Ptph-1::GFP* |
| IZ2277 | *ufEx802* | *Pckr-1::genomic ckr-1 [pDT112 (100ng/µL)]*  *+ pHP6 [Plgc-11::GFP (50ng/µL)]* |
| IZ2399 | *nlp-12(ok335)I; ufEx802* |  |
| IZ3201 | *ufEx953* | *Prgef-1::ckr-1 minigene [pRB20 (25ng/µL)]*  *+ Plgc-11::GFP (50ng/µL)* |
| IZ3217 | *ufEx689* | *Pckr-1::ckr-1 minigene [pDT231 (20ng/µL)]*  *+ Punc-122::GFP (20ng/µL)]* |
| IZ3198 | *ufEx962* | *Pmyo-3::ckr-1 minigene [pRB16 (25ng/µL)]*  *+ Pelt-2::GFP (50ng/µL)* |
| IZ3211 | *ufEx1309* | *Punc-17β::ckr-1 minigene [pRB14 (50ng/µL)]*  *+ Plgc-11::GFP [pHP6 (50ng/µL)]* |
| IZ3212 | *ufEx1310* | *Punc-47::ckr-1 minigene [pRB15(25ng/µL)]*  *+ Plgc-11::GFP [pHP6 (50 ng/µL)]* |
| IZ3197 | *ufEx930* | *Plgc-55::ckr-1 minigene [pRB17(25 ng/µL)]*  *+ pHP6 (50 ng/µL)]* |
| IZ3203 | *ufEx1003* | *Podr-2(16)::ckr-1 minigene [pRB27(25ng/µL)]*  *+ Plgc-11::GFP [pHP6 (50ng/µL)]* |

| IZ3650 | *ufEx1538* | *Pflp-22(∆4)::ckr-1 minigene [pSR37(50 ng/µL)] + Plgc-11::GFP [pHP6 (50 ng/µL)]* |
| --- | --- | --- |
| IZ3210 | *ufEx1180* | *Pgcy-28d::ckr-1 minigene [pRB29 (25 ng/µL)] + Plgc-11::GFP [pHP6 (50 ng/µL)]* |
| IZ3199 | *ufEx1181* | *Podr-2(18)::ckr-1 minigene [pRB26 (25 ng/µL)] + Plgc-11::GFP [ pHP6 (50 ng/µL)]* |
| IZ3231 | *ufEx1196* | *Posm-6::ckr-1 minigene [pNB66 (25 ng/µL)]*  *+ Plgc-11::mCherry [pBB107 (50 ng/µL)]* |
| IZ3200 | *ufEx1197* | *Plim-4::ckr-1 minigene [pNB67 (25 ng/µL)]*  *+ Plgc-11::mCherry [pBB107 (50 ng/µL)]* |
| IZ3222 | *ufEx1234* | *Pnpr-9::ckr-1 minigene [pNB61 (25 ng/µL)]*  *+ Plgc-11::GFP [pHP6 (80 ng/µL)]* |
| IZ2461 | *ckr-1(ok2502)I; ufEx911* | *Pckr-1::ckr-1 [pDT231 (5 ng/µL)]*  *+ Plgc-11::GFP [pHP6 (50 ng/µL)]* |
| IZ3112 | *ckr-1(ok2502) I; ufEx1247* | *Podr-2(16)::ckr-1 minigene (5ng/µL)*  *+ Plgc-11::GFP [pHP6 (80 ng/µL) ]* |
| IZ3116 | *ckr-1(ok2502) I; ufEx1250* | *Plgc-55::ckr-1 minigene [pRB17 (5ng/µL)]*  *+ Plgc-11::GFP [pHP6 (80 ng/µL)]* |
| IZ3890 | *ckr-1(ok2502) I; ufEx1646* | *Pflp-22∆4::ckr-1 minigene [pSR33 (5 ng/µL)]*  *+ Punc122::GFP (50ng/µL)* |
| IZ3875 | *ufEx1638* | *Pflp-22∆4::ckr-1 minigene [pSR33 (5 ng/µL)]*  *+ Punc-122::RFP (50 ng/µL)* |
| IZ3587 | *ufEx1518* | *Pflp-22(∆4)::miniSOG, Pflp-22(∆4)::GFP [pSR19D (50ng/µL) + pSR17A (50 ng/µL)]*  *+ Plgc-11::mCherry [pBB107 (50 ng/µL)]* |
| IZ3701 | *ufEx802*; *ufEx1518* | *Pckr-1::ckr-1* genomic; *Pflp-22(∆4)::miniSOG; Pflp-22∆4::GFP* |
| IZ1782 | *lite-1(ce314) X; ufIs140* | *Pnlp-12::ChR::GFP [pCL28 (50 ng/µL)]*  *+ Plgc-11::GFP [pHP6 (30 ng/µL)]* |
| IZ1779 | *nlp-12(ok335) I; lite-1(ce314) X; ufIs140* |  |
| IZ1968 | *ckr-1(ok2502) I; ckr-2(tm3082) III;*  *lite-1(ce314) X; ufIs140* |  |
| IZ1777 | *ckr-1(ok2502) I; lite-1(ce314) X; ufIs140* |  |
| IZ1781 | *ckr-2(tm3082) III; lite-1(ce314) X; ufIs140* |  |
| IZ3645 | *ufIs186* | *Podr-2(16)::Chrimson [pSR11 (50 ng/µL]; Punc-122::GFP (50 ng/µL)* |
| IZ3598 | *ufEx1522* | *Pflp-22∆4::HisCl1::SL2::GFP [pSR20 (50 ng/µL)]; Plgc-11::mCherry [pBB107 (50 ng/µL)]* |
| IZ3788 | *ufEx1584* | *Pflp-22∆4::GCaMP6s::SL2::mCherry [pSR26 (50 ng/µL)]; Punc-122::GFP (50ng/µL)* |
| IZ4208 | *lin-15(n765ts)X; ufEx1784* | *Pflp-22∆4::GCaMP6s::SL2::mCherry*  *[pSR26 (50 ng/µL)]; Plin-15::lin-15+ [pL15EK (50ng/µL)]* |
| IZ3824 | *ufEx1584; ckr-1(ok2502) I* |  |
| IZ4164 | *ufEx1759; ckr-1(ok2502) I* | *Pckr-2::ckr-1 minigene [pSR81 (5 ng/µL)]; Pinx-6::GFP [pDO125 (50 ng/mL)]* |
| IZ4255 | *ufEx1815; nlp-12(lf) I* | *Pser-2(prom3)::nlp-12 [pCL131 (5 ng/µL)]; Plgc-11::mCherry[pBB107 (50ng/µL)]* |
