# Supplementary material for: A conserved neuropeptide system links head and body motor circuits to enable adaptive behavior
Source: eLife. 2021 Nov 12;10:e71747. doi: 10.7554/eLife.71747 (PMC8626090; doi:10.7554/eLife.71747)
Supplement: Supplementary file 2. — * Indicated strains were crossed into ufIs141 (Pckr-1::ckr-1::SL2::GFP) to generate strains to determine colocalization. #+ or – indicates presence or absence of ckr-1 expression in identified neuron. * Indicated strains were crossed into ufIs141 to generate strains to determine colocalization, #+ indicates ckr-1 expression, - indicates absence. [file elife-71747-supp2.docx]

**Supplementary File 2**

Identification (method of ID, marker and strain indicated for each neuron) to determine *ckr-1* expressing neurons. * Indicated strains were crossed into *ufIs141* *(Pckr-1::ckr-1::SL2::GFP)* to generate strains to determine colocalization. ^#^ + or – indicates presence or absence of *ckr-1* expression in identified neuron.

| **Neuron type** | **Neuron class** | ***ckr-1* #** | **Method of identification** | **Marker** | **Strain*** | **Reference** |
| --- | --- | --- | --- | --- | --- | --- |
| Sensory | ASK | + | DiI staining |  |  |  |
|  | ASI | + | DiI staining |  |  |  |
|  | AWB | + | DiI staining |  |  |  |
|  | ASH | + | DiI staining |  |  |  |
|  | PHA | + | Dye uptake |  |  |  |
|  | PHB | + | Dye uptake |  |  |  |
|  | NSM (motor+ sensory) | + | Anatomical + colocalization* | *Ptph-1::GFP* | *mgIs42* | (Sze et al., 2000) |
|  | IL1, IL2 | - |  |  |  |  |
|  | OLQ, OLV | - |  |  |  |  |
|  | BAG | - |  |  |  |  |
| Interneuron | AIA (Ach) | + | Colocalization* | *Psra-11::GFP* | *otIs123* | (Altun-Gultekin et al., 2001) |
|  | AIY | - | Colocalization* | *Pttx-3::GFP* | *mgIs18* | (Hobert et al., 1997) |
|  | AIB (Ach) | + |  | *Podr-2(2b)::GFP* | *kyIs51* | (Chou et al., 2001) |
|  | AVL (GABA) | + | Colocalization* | *Punc-47::GFP* | *oxIs12* | (McIntire et al., 1997) |
|  | RIS (GABA) | + | Colocalization* | *Punc-47::GFP* | *oxIs12* |  |
|  | RIG (glutamate) | + | Colocalization* | *Podr-2(18)::GFP* | *ufEx863* | (Chou et al., 2001) |
|  | RIS | + | Colocalization* | *Pser-4::GFP* | *adEx1616* | (Gürel et al., 2012) |
|  | PVQ | + | Colocalization* | *Psra-6::GFP* | *oyIs14* | (Aurelio et al., 2003) |
| Head motor | RMEV/D | + | Colocalization* | *Punc-47::GFP* | *oxIs12* |  |
|  | SMDV/D | + | Colocalization* | *Plad-2::GFP*  *Plgc-55::GFP*  *Podr-2(16)::GFP*  *Pflp-22(∆4)::GFP* | *otIs337*  *zfIs6*  *ufEx1485*  *ufEx1504* | (Wang et al., 2008)  (Pirri et al., 2009)  (Chou et al., 2001)  (Yeon et al., 2018) |
|  | RIV | + | Colocalization* | *Pnpr-4::RFP* | *dbEx721* | (Cohen et al., 2009) |
| Ventral cord motor | Cholinergic  VA/VB/DA/DB | + | Colocalization* | *Punc-17::GFP*  *Pacr-2::mCherry* | *vsIs48*  *ufIs43* | (Alfonso et al., 1993)  (Petrash et al., 2013) |
|  | GABAergic | - | Colocalization* | *Punc-47::GFP* | *oxIs12* |  |

* Indicated strains were crossed into *ufIs141* to generate strains to determine colocalization

# + indicates *ckr-1* expression, - indicates absence

**References**

Alfonso A, Grundahl K, Duerr J, Han H, Rand J. 1993. The Caenorhabditis elegans unc-17 gene: a putative vesicular acetylcholine transporter. *Science* 261:617–619. doi:10.1126/science.8342028

Altun-Gultekin Z, Andachi Y, Tsalik EL, Pilgrim D, Kohara Y, Hobert O. 2001. A regulatory cascade of three homeobox genes, ceh-10, ttx-3 and ceh-23, controls cell fate specification of a defined interneuron class in C. elegans. *Dev Camb Engl* 128:1951–69.

Aurelio O, Boulin T, Hobert O. 2003. Identification of spatial and temporal cues that regulate postembryonic expression of axon maintenance factors in the C. elegans ventral nerve cord. *Development* 130:599–610. doi:10.1242/dev.00277

Chou JH, Bargmann CI, Sengupta P. 2001. The Caenorhabditis elegans odr-2 gene encodes a novel Ly-6-related protein required for olfaction. *Genetics* 157:211–24.

Cohen M, Reale V, Olofsson B, Knights A, Evans P, Bono M de. 2009. Coordinated Regulation of Foraging and Metabolism in C. elegans by RFamide Neuropeptide Signaling. *Cell Metab* 9:375–385. doi:10.1016/j.cmet.2009.02.003

Gürel G, Gustafson MA, Pepper JS, Horvitz HR, Koelle MR. 2012. Receptors and Other Signaling Proteins Required for Serotonin Control of Locomotion in Caenorhabditis elegans. *Genetics* 192:1359–1371. doi:10.1534/genetics.112.142125

Hobert O, Mori I, Yamashita Y, Honda H, Ohshima Y, Liu Y, Ruvkun G. 1997. Regulation of Interneuron Function in the C. elegans Thermoregulatory Pathway by the ttx-3 LIM Homeobox Gene. *Neuron* 19:345–357. doi:10.1016/s0896-6273(00)80944-7

McIntire SL, Reimer RJ, Schuske K, Edwards RH, Jorgensen EM. 1997. Identification and characterization of the vesicular GABA transporter. *Nature* 389:870–876. doi:10.1038/39908

Petrash HA, Philbrook A, Haburcak M, Barbagallo B, Francis MM. 2013. ACR-12 Ionotropic Acetylcholine Receptor Complexes Regulate Inhibitory Motor Neuron Activity in Caenorhabditis elegans. *J Neurosci* 33:5524–5532. doi:10.1523/jneurosci.4384-12.2013

Pirri JK, McPherson AD, Donnelly JL, Francis MM, Alkema MJ. 2009. A Tyramine-Gated Chloride Channel Coordinates Distinct Motor Programs of a Caenorhabditis elegans Escape Response. *Neuron* 62:526–538. doi:10.1016/j.neuron.2009.04.013

Sze JY, Victor M, Loer C, Shi Y, Ruvkun G. 2000. Food and metabolic signalling defects in a Caenorhabditis elegans serotonin-synthesis mutant. *Nature* 403:560–564. doi:10.1038/35000609

Wang X, Zhang W, Cheever T, Schwarz V, Opperman K, Hutter H, Koepp D, Chen L. 2008. The C. elegans L1CAM homologue LAD-2 functions as a coreceptor in MAB-20/Sema2 mediated axon guidance. *J Cell Biology* 180:233–46. doi:10.1083/jcb.200704178

Yeon J, Kim Jinmahn, Kim D-Y, Kim H, Kim Jungha, Du EJ, Kang K, Lim H-H, Moon D, Kim K. 2018. A sensory-motor neuron type mediates proprioceptive coordination of steering in C. elegans via two TRPC channels. *Plos Biol* 16:e2004929. doi:10.1371/journal.pbio.2004929
