# Supplementary material for: A conserved neuropeptide system links head and body motor circuits to enable adaptive behavior
Source: eLife. 2021 Nov 12;10:e71747. doi: 10.7554/eLife.71747 (PMC8626090; doi:10.7554/eLife.71747)
Supplement: Supplementary file 3. — **Bold indicates neurons where ckr-1 is expressed. [file elife-71747-supp3.docx]

| **Promoter** | **Expression** | **References** |
| --- | --- | --- |
| *Pckr-1* | *ckr-1* native promoter (3564 bp) |  |
| *Prgef-1* | Pan-neuronal | (Chen et al., 2011) |
| *Punc-17β* | **Restricted expression in cholinergic motor neurons (VNC)** | (Charlie et al., 2006) |
| *Punc-47* | All GABAergic neurons | (McIntire et al., 1997) |
| *Pmyo-3* | Muscles | (Okkema et al., 1993) |
| *Podr-2(16)* | **SMD, RME** | (Chou et al., 2001) |
| *Plgc-55* | **SMD**, IL1, AVB, RMD, neck muscles | (Pirri et al., 2009) |
| *Pflp-22∆4* | **SMD**, URX | (Yeon et al., 2018) |
| *Pgcy-28.d* | **AIA** | (Shinkai et al., 2011) |
| *Plim-4(s)* (lim-4 promoter fragment from -3328 to -2174 upstream of start) | **RIV** | (Pirri et al., 2009) |
| *Pnpr-9* | **AIB** | (Campbell et al., 2016) |
| *Podr-2(18)* | **RIG**, SMB | (Chou et al., 2001) |
| *Posm-6* | ADE, **amphid neurons**, AQR, CEM, CEP, HOA, HOB, IL1, IL2, OLL, OLQ, PDE, **phasmid neurons**. | (Collet et al., 1998) |

**Supplementary File 3**

Promoters used in *ckr-1(OE)* screen (Fig. 5C) indicating expression pattern. ****Bold** indicates neurons where *ckr-1* is expressed.

**References**

Campbell JC, Polan-Couillard LF, Chin-Sang ID, Bendena WG. 2016. NPR-9, a Galanin-Like G-Protein Coupled Receptor, and GLR-1 Regulate Interneuronal Circuitry Underlying Multisensory Integration of Environmental Cues in Caenorhabditis elegans. *Plos Genet* 12:e1006050. doi:10.1371/journal.pgen.1006050

Charlie NK, Schade MA, Thomure AM, Miller KG. 2006. Presynaptic UNC-31 (CAPS) Is Required to Activate the Gαs Pathway of the Caenorhabditis elegans Synaptic Signaling Network. *Genetics* 172:943–961. doi:10.1534/genetics.105.049577

Chen L, Fu Y, Ren M, Xiao B, Rubin CS. 2011. A RasGRP, C. elegans RGEF-1b, Couples External Stimuli to Behavior by Activating LET-60 (Ras) in Sensory Neurons. *Neuron* 70:51–65. doi:10.1016/j.neuron.2011.02.039

Chou JH, Bargmann CI, Sengupta P. 2001. The Caenorhabditis elegans odr-2 gene encodes a novel Ly-6-related protein required for olfaction. *Genetics* 157:211–24.

Collet J, Spike CA, Lundquist EA, Shaw JE, Herman RK. 1998. Analysis of osm-6, a gene that affects sensory cilium structure and sensory neuron function in Caenorhabditis elegans. *Genetics* 148:187–200.

McIntire SL, Reimer RJ, Schuske K, Edwards RH, Jorgensen EM. 1997. Identification and characterization of the vesicular GABA transporter. *Nature* 389:870–876. doi:10.1038/39908

Okkema PG, Harrison SW, Plunger V, Aryana A, Fire A. 1993. Sequence requirements for myosin gene expression and regulation in Caenorhabditis elegans. *Genetics* 135:385–404. doi:10.1093/genetics/135.2.385

Pirri JK, McPherson AD, Donnelly JL, Francis MM, Alkema MJ. 2009. A Tyramine-Gated Chloride Channel Coordinates Distinct Motor Programs of a Caenorhabditis elegans Escape Response. *Neuron* 62:526–538. doi:10.1016/j.neuron.2009.04.013

Shinkai Y, Yamamoto Y, Fujiwara M, Tabata T, Murayama T, Hirotsu T, Ikeda DD, Tsunozaki M, Iino Y, Bargmann CI, Katsura I, Ishihara T. 2011. Behavioral Choice between Conflicting Alternatives Is Regulated by a Receptor Guanylyl Cyclase, GCY-28, and a Receptor Tyrosine Kinase, SCD-2, in AIA Interneurons of Caenorhabditis elegans. *J Neurosci* 31:3007–3015. doi:10.1523/jneurosci.4691-10.2011

Yeon J, Kim Jinmahn, Kim D-Y, Kim H, Kim Jungha, Du EJ, Kang K, Lim H-H, Moon D, Kim K. 2018. A sensory-motor neuron type mediates proprioceptive coordination of steering in C. elegans via two TRPC channels. *Plos Biol* 16:e2004929. doi:10.1371/journal.pbio.2004929
