# Supplementary material for: A conserved neuropeptide system links head and body motor circuits to enable adaptive behavior
Source: eLife. 2021 Nov 12;10:e71747. doi: 10.7554/eLife.71747 (PMC8626090; doi:10.7554/eLife.71747)
Supplement: Supplementary file 4. — For cell specific overexpression or rescue of ckr-1, ckr-1 minigene was expressed under indicated promoters. Entry vectors containing promoters recombined with destination vectors pRB12 or pRB13 for cell-specific overexpression or rescue of ckr-1. [file elife-71747-supp4.docx]

**Supplementary File 4**

Plasmid constructs used in cell specific *ckr-1(OE)* screen or cell-specific rescue (Fig. 5C, 7A). For cell specific overexpression or rescue of *ckr-1*, *ckr-1* minigene was expressed under indicated promoters. Entry vectors containing promoters recombined with destination vectors pRB12 or pRB13 for cell-specific overexpression or rescue of *ckr-1*.

| **Plasmid** | ***ckr-1* expression construct** | **Reference** |
| --- | --- | --- |
| pRB31 | *Pacr-2::ckr-1* | (Petrash et al., 2013) |
| pRB14 | *Punc-17β::ckr-1* | (Charlie et al., 2006) |
| pRB16 | *Pmyo-3::ckr-1* | (Okkema et al., 1993) |
| pRB30 | *Plad-2::ckr-1* | (Wang et al., 2008) |
| pRB17 | *Plgc-55::ckr-1* | (Pirri et al., 2009) |
| pRB27 | *Podr-2(16)::ckr-1* | (Chou et al., 2001) |
| pSR33 | *Pflp-22∆4::ckr-1* | (Yeon et al., 2018) |
| pRB18 | *Pglr-2::ckr-1* | (Brockie et al., 2001) |
| pRB20 | *PF25B3.3::ckr-1* | (Chen et al., 2011) |
| pRB21 | *Pttr-39::ckr-1* | (Petersen et al., 2011) |
| pRB22 | *Pdel-1::ckr-1* | (Tavernarakis et al., 1997) |
| pRB23 | *Punc-129::ckr-1* | (Colavita et al., 1998) |
| pRB24 | *Plim-6 Intron 4::ckr-1* | (Turek et al., 2013) |
| pRB26 | *Podr-2(18)::ckr-1* | (Chou et al., 2001) |
| pRB28 | *Ptph-1::ckr-1* | (Sze et al., 2000) |
| pRB29 | *Pgcy-28d::ckr-1* | (Shinkai et al., 2011) |
| pNB66 | *Posm-6::ckr-1* | (Collet et al., 1998) |
| pNB67 | *Plim-4::ckr-1* | (Pirri et al., 2009) |
| pNB61 | *Pnpr-9::ckr-1* | (Campbell et al., 2016) |

**References**

Brockie PJ, Madsen DM, Zheng Y, Mellem J, Maricq AV. 2001. Differential Expression of Glutamate Receptor Subunits in the Nervous System of Caenorhabditis elegans and Their Regulation by the Homeodomain Protein UNC-42. *J Neurosci* 21:1510–1522. doi:10.1523/jneurosci.21-05-01510.2001

Campbell JC, Polan-Couillard LF, Chin-Sang ID, Bendena WG. 2016. NPR-9, a Galanin-Like G-Protein Coupled Receptor, and GLR-1 Regulate Interneuronal Circuitry Underlying Multisensory Integration of Environmental Cues in Caenorhabditis elegans. *Plos Genet* 12:e1006050. doi:10.1371/journal.pgen.1006050

Charlie NK, Schade MA, Thomure AM, Miller KG. 2006. Presynaptic UNC-31 (CAPS) Is Required to Activate the Gαs Pathway of the Caenorhabditis elegans Synaptic Signaling Network. *Genetics* 172:943–961. doi:10.1534/genetics.105.049577

Chen L, Fu Y, Ren M, Xiao B, Rubin CS. 2011. A RasGRP, C. elegans RGEF-1b, Couples External Stimuli to Behavior by Activating LET-60 (Ras) in Sensory Neurons. *Neuron* 70:51–65. doi:10.1016/j.neuron.2011.02.039

Chou JH, Bargmann CI, Sengupta P. 2001. The Caenorhabditis elegans odr-2 gene encodes a novel Ly-6-related protein required for olfaction. *Genetics* 157:211–24.

Colavita A, Krishna S, Zheng H, Padgett RW, Culotti JG. 1998. Pioneer axon guidance by UNC-129, a C. elegans TGF-beta. *Sci New York N Y* 281:706–9. doi:10.1126/science.281.5377.706

Collet J, Spike CA, Lundquist EA, Shaw JE, Herman RK. 1998. Analysis of osm-6, a Gene That Affects Sensory Cilium Structure and Sensory Neuron Function in Caenorhabditis elegans. *Genetics* 148:187–200. doi:10.1093/genetics/148.1.187

Okkema PG, Harrison SW, Plunger V, Aryana A, Fire A. 1993. Sequence requirements for myosin gene expression and regulation in Caenorhabditis elegans. *Genetics* 135:385–404. doi:10.1093/genetics/135.2.385

Petersen SC, Watson JD, Richmond JE, Sarov M, Walthall WW, Miller DM. 2011. A Transcriptional Program Promotes Remodeling of GABAergic Synapses in Caenorhabditis elegans. *J Neurosci* 31:15362–15375. doi:10.1523/jneurosci.3181-11.2011

Petrash HA, Philbrook A, Haburcak M, Barbagallo B, Francis MM. 2013. ACR-12 Ionotropic Acetylcholine Receptor Complexes Regulate Inhibitory Motor Neuron Activity in Caenorhabditis elegans. *J Neurosci* 33:5524–5532. doi:10.1523/jneurosci.4384-12.2013

Pirri JK, McPherson AD, Donnelly JL, Francis MM, Alkema MJ. 2009. A tyramine-gated chloride channel coordinates distinct motor programs of a Caenorhabditis elegans escape response. *Neuron* 62:526–38. doi:10.1016/j.neuron.2009.04.013

Shinkai Y, Yamamoto Y, Fujiwara M, Tabata T, Murayama T, Hirotsu T, Ikeda DD, Tsunozaki M, Iino Y, Bargmann CI, Katsura I, Ishihara T. 2011. Behavioral Choice between Conflicting Alternatives Is Regulated by a Receptor Guanylyl Cyclase, GCY-28, and a Receptor Tyrosine Kinase, SCD-2, in AIA Interneurons of Caenorhabditis elegans. *J Neurosci* 31:3007–3015. doi:10.1523/jneurosci.4691-10.2011

Sze JY, Victor M, Loer C, Shi Y, Ruvkun G. 2000. Food and metabolic signalling defects in a Caenorhabditis elegans serotonin-synthesis mutant. *Nature* 403:560–564. doi:10.1038/35000609

Tavernarakis N, Shreffler W, Wang S, Driscoll M. 1997. unc-8, a DEG/ENaC Family Member, Encodes a Subunit of a Candidate Mechanically Gated Channel That Modulates C. elegans Locomotion. *Neuron* 18:107–119. doi:10.1016/s0896-6273(01)80050-7

Turek M, Lewandrowski I, Bringmann H. 2013. An AP2 Transcription Factor Is Required for a Sleep-Active Neuron to Induce Sleep-like Quiescence in C. elegans. *Curr Biol* 23:2215–2223. doi:10.1016/j.cub.2013.09.028

Wang X, Zhang W, Cheever T, Schwarz V, Opperman K, Hutter H, Koepp D, Chen L. 2008. The C. elegans L1CAM homologue LAD-2 functions as a coreceptor in MAB-20/Sema2 mediated axon guidance. *J Cell Biology* 180:233–46. doi:10.1083/jcb.200704178

Yeon J, Kim Jinmahn, Kim D-Y, Kim H, Kim Jungha, Du EJ, Kang K, Lim H-H, Moon D, Kim K. 2018. A sensory-motor neuron type mediates proprioceptive coordination of steering in C. elegans via two TRPC channels. *Plos Biol* 16:e2004929. doi:10.1371/journal.pbio.2004929
