# Supplementary material for: A conserved neuropeptide system links head and body motor circuits to enable adaptive behavior
Source: eLife. 2021 Nov 12;10:e71747. doi: 10.7554/eLife.71747 (PMC8626090; doi:10.7554/eLife.71747)
Supplement: Supplementary file 5. [file elife-71747-supp5.docx]

**Supplementary File 5**

Promoter lengths and primer information for promoters used

| *Pckr-1*  3564bp promoter region of *ckr-1* amplified from genomic DNA  OMF2159 (Forward primer): CTGCAGGATGGAGATTCAATCAGC  OMF2160 (Reverse primer): TGTGTATCTGAAAATTTTTAATTTTAAA |
| --- |
| *Pckr-2*  8.6 kb promoter region of *ckr-2* amplified from genomic DNA  OMF1067 (Forward primer): GCATGCGGGTGTGATAAGTGCAATGAAGTGG  OMF1068 (Reverse primer): ACGTACCGGTTCCTCCTGATGTACCGTTGACATTGTGG |
| *Plgc-55* 2663bp amplified from plasmid lgc55_2663-0_pPD95_  OMF1019 (Forward primer): ATGTCTGCCCCTATCACCAGTG  OMF1020 (Reverse primer): TTCATTTCGACATCTATTTGCCAATA  (Pirri et al., 2009) |
| *Podr-2(16)*  3.2 kb promoter fragment amplified from genomic DNA  OMF1878 (Forward primer): ATGGGAATGGCGGCAAAT  OMF1879 (Reverse primer): CGGGCATCCCGACAAACTGT  (Chou et al., 2001) |
| *Plad-2*  4.5 kb upstream of *lad-2* ATG amplified from genomic DNA  OMF1876 (Forward primer): ATTTTTTGCTGTGTTCCACTAA  OMF1920 (Reverse primer): TGTTGGAAAAATCCAAAAAAAAAGTCTGC  (Wang et al., 2008) |
| *Pflp-22(∆4)*  1532bp promoter fragment was amplified from genomic DNA*.*  OMF2610 (Forward primer): TGCAGGAAACACCACCTAGTATATAAT  OMF2611 (Reverse primer): TGCAAGCTTAGAGTACAACGGCGA  (Yeon et al., 2018) |
| *Pmyo-3*  Amplified myo-3 promoter(2344bp) from pBB38 (*Pmyo-3::acr-2 cDNA*)  OMF763 (Forward primer): CGGCTATAATAAGTTCTTGAATAA  OMF764 (Reverse primer): CCTGAAAATTAGACGGTAAAAGTG  (Okkema et al., 1993) |
| *Punc17β*  *unc-17β promoter (485bp) amplified from plasmid pRM#621*  OMF1594 (Forward primer): TTGGTTTTCACAATTTTCTGGTTTT  OMF1595 (Reverse primer): TTGAACAAGAGATGCGGAAAATAGAAAGA  (Charlie et al., 2006) |
| *Punc-47 unc-47* promoter (1158bp) amplified from genomic DNA  OMF658 (Forward primer): AGTCGAAAGTCGGTGGCAAG  OMF659 (Reverse primer): GTCGTCTCACAGGAAAGACAGAT  (McIntire et al., 1997) |
| *Podr-2(18)*  2.4 kb promoter fragment amplified from genomic DNA  OMF1015 (Forward primer): GAACAGGGTCTCTCACAGTTTGTCG  OMF1016 (Reverse primer): CCATCAGCCAAATGTAGGCTCGG  (Chou et al., 2001) |
| *Pnpr-9  npr-9* promoter (2305bp) amplified from genomic DNA  OMF1353 (Forward primer): CAATGCAGAAGAAGACTCTTCATCC  OMF1544 (Reverse primer): GACATTTCCCAACGACATTTCCC  (Altun-Gultekin et al., 2001) |
| *Pgcy-28d*  2841 bp promoter for *gcy-28d* amplified from genomic DNA.  OMF1884 (Forward primer): TACAATTGTAGTGAGCTTCG  OMF1885 (Reverse primer): TTCGCACTCATCTCACCATTCC  (Shinkai et al., 2011) |
| *Plim-4*  *lim-4* promoter fragment from -3328 to -2174 upstream of start was amplified from plasmid  [*Plim-4(-3328 to -2174)::NLSwCherry::SL2::GCaMP6s]*  OMF2166 (Forward primer): AAGCTTTGATTTAGAAATTGTAGTTTC  OMF2167 (Reverse primer): ACAAGCCGCTCAGTTTTGATCTAAAAT  (Pirri et al., 2009) |

| *Posm-6*  2083 bp promoter region from *Posm-6::GFP*  OMF2227 (Forward primer): CTTGCATGTTATGGATACTCTGAAT  OMF2228 (Reverse primer): TTTTCGTCTGAAAATGGAGGCATAG  (Collet et al., 1998) |
| --- |
| *Ptph-1*  *tph-1* promoter was amplified from *Ptph-1::unc-2*  OMF926 (Forward primer): GGTGGTCTTCCCGCTTGCAATAC  OMF927 (Reverse primer): ATGATTGAAGAGAGCAATGCTAC  (Sze et al., 2000) |
| *Pser-2prom3*  4101 bp promoter region of the *ser-*2 promoter (*ser-2prom3*) was amplified from genomic DNA  OMF3149 Forward primer: GTAAAAGTTTAGTAAATTAACTGCTA  OMF3150 Reverse primer: TATGTGTTGTGATGTCACAAAAATATG  (Tao et al., 2019) |

**References**

Altun-Gultekin Z, Andachi Y, Tsalik EL, Pilgrim D, Kohara Y, Hobert O. 2001. A regulatory cascade of three homeobox genes, ceh-10, ttx-3 and ceh-23, controls cell fate specification of a defined interneuron class in C. elegans. *Dev Camb Engl* 128:1951–69.

Charlie NK, Schade MA, Thomure AM, Miller KG. 2006. Presynaptic UNC-31 (CAPS) Is Required to Activate the Gαs Pathway of the Caenorhabditis elegans Synaptic Signaling Network. *Genetics* 172:943–961. doi:10.1534/genetics.105.049577

Chou JH, Bargmann CI, Sengupta P. 2001. The Caenorhabditis elegans odr-2 gene encodes a novel Ly-6-related protein required for olfaction. *Genetics* 157:211–24.

Collet J, Spike CA, Lundquist EA, Shaw JE, Herman RK. 1998. Analysis of osm-6, a gene that affects sensory cilium structure and sensory neuron function in Caenorhabditis elegans. *Genetics* 148:187–200.

McIntire SL, Reimer RJ, Schuske K, Edwards RH, Jorgensen EM. 1997. Identification and characterization of the vesicular GABA transporter. *Nature* 389:870–876. doi:10.1038/39908

Okkema PG, Harrison SW, Plunger V, Aryana A, Fire A. 1993. Sequence requirements for myosin gene expression and regulation in Caenorhabditis elegans. *Genetics* 135:385–404. doi:10.1093/genetics/135.2.385

Pirri JK, McPherson AD, Donnelly JL, Francis MM, Alkema MJ. 2009. A Tyramine-Gated Chloride Channel Coordinates Distinct Motor Programs of a Caenorhabditis elegans Escape Response. *Neuron* 62:526–538. doi:10.1016/j.neuron.2009.04.013

Shinkai Y, Yamamoto Y, Fujiwara M, Tabata T, Murayama T, Hirotsu T, Ikeda DD, Tsunozaki M, Iino Y, Bargmann CI, Katsura I, Ishihara T. 2011. Behavioral Choice between Conflicting Alternatives Is Regulated by a Receptor Guanylyl Cyclase, GCY-28, and a Receptor Tyrosine Kinase, SCD-2, in AIA Interneurons of Caenorhabditis elegans. *J Neurosci* 31:3007–3015. doi:10.1523/jneurosci.4691-10.2011

Sze JY, Victor M, Loer C, Shi Y, Ruvkun G. 2000. Food and metabolic signalling defects in a Caenorhabditis elegans serotonin-synthesis mutant. *Nature* 403:560–564. doi:10.1038/35000609

Tao L, Porto D, Li Z, Fechner S, Lee SA, Goodman MB, Xu XZS, Lu H, Shen K. 2019. Parallel Processing of Two Mechanosensory Modalities by a Single Neuron in C. elegans. *Dev Cell*. doi:10.1016/j.devcel.2019.10.008

Wang X, Zhang W, Cheever T, Schwarz V, Opperman K, Hutter H, Koepp D, Chen L. 2008. The C. elegans L1CAM homologue LAD-2 functions as a coreceptor in MAB-20/Sema2 mediated axon guidance. *J Cell Biology* 180:233–46. doi:10.1083/jcb.200704178

Yeon J, Kim Jinmahn, Kim D-Y, Kim H, Kim Jungha, Du EJ, Kang K, Lim H-H, Moon D, Kim K. 2018. A sensory-motor neuron type mediates proprioceptive coordination of steering in C. elegans via two TRPC channels. *Plos Biol* 16:e2004929. doi:10.1371/journal.pbio.2004929
